# Plasmonic Gold Decorated MWCNT Nanocomposite for Localized Plasmon Resonance Sensing

**DOI:** 10.1038/srep13181

**Published:** 2015-08-18

**Authors:** J. Ozhikandathil, S. Badilescu, M. Packirisamy

**Affiliations:** 1 Concordia University, Optical Bio-Microsystems Laboratory, Department of Mechanical and Industrial Engineering, Concordia University, Montreal, H3G 1M8, Canada

## Abstract

The synergism of excellent properties of carbon nanotubes and gold nanoparticles is used in this work for bio-sensing of recombinant bovine growth hormones (rbST) by making Multi Wall Carbon Nanotubes (MWCNT) locally optically responsive by augmenting it optical properties through Localized Surface Plasmon Resonance (LSPR). To this purpose, locally gold nano particles decorated gold–MWCNT composite was synthesized from a suspension of MWCNT bundles and hydrogen chloroauric acid in an aqueous solution, activated ultrasonically and, then, drop-casted on a glass substrate. The slow drying of the drop produces a “coffee ring” pattern that is found to contain gold–MWCNT nanocomposites, accumulated mostly along the perimeter of the ring. The reaction is studied also at low-temperature, in the vacuum chamber of the Scanning Electron Microscope and is accounted for by the local melting processes that facilitate the contact between the bundle of tubes and the gold ions. Biosensing applications of the gold–MWCNT nanocomposite using their LSPR properties are demonstrated for the plasmonic detection of traces of bovine growth hormone. The sensitivity of the hybrid platform which is found to be 1 ng/ml is much better than that measuring with gold nanoparticles alone which is only 25 ng/ml.

Chip-based plasmonic platforms are using the Localized Surface Plasmon Resonance (LSPR) of gold nanostructures immobilized on a solid substrate and, in this way, measurements can be done in the transmission mode, by using a UV-Visible spectrophotometer. Gold nanostructures alone and gold – polymer nanocomposites have been successfully used by us^1^,^2^,^3^,^4^ and other^5^,^6^,^7^ groups as very sensitive platforms for the detection of biomolecules. On the other hand, biosensors based on Carbon Nanotubes (CNTs) alone, in particular, field effect transistors based on semiconducting CNTs have been successfully used for electrochemical detection of protein binding^8^,^9^. It has been shown that the sensitivity of the electrical detection of protein binding was enhanced by introducing the Au nanoparticle-antibody conjugates in a carbon nanotube field effect transistor^10^. Due to the large surface-to-volume ratio and the high electron mobility of CNTs, they have been used with very good results as sensing material for gas sensing as well and the sensitivity toward gases was found to be increased in the presence of Au nanoclusters^11^,^12^. CNTs have also been widely demonstrated for the electrochemical detection of glucose in a very low concentration as the presence of biomolecules will trigger a change in the electrical properties of CNTs. Due to the synergetic contributions of the two components, Au-Multi Wall Carbon Nanotubes (MWCNT) nanocomposite possess valuable properties that are amenable to sensing applications. While CNTs are retaining their electrical behavior, gold nanoparticles may introduce new attachment sites for implementing Localized Surface Plasmon Resonance (LSPR) phenomenon, important for the sensing capabilities of the Au-MWCNT hybrids. The integration of gold particles into the MWCNT bundles opens up new opportunities for the ultrasensitive detection of biomolecules, either electrically or optically, by using the plasmonic properties of gold. Until now, carbon nanotubes were used only to enhance the surface plasmon resonance signal of gold-coated glass plate of the Biacore biosensing systems^13^,^14^. Moreover, recently, the plasmonic coupling of CNT forests and Au nanoparticles was used for SERS sensing^15^. In the present work, a novel method of synthesizing the Au-MWCNT hybrid composite and deposition to a glass substrate by drop casting to a coffee ring pattern is explored to make MWCNT bundles to become locally sensitive for LSPR properties which opens up a huge potential for expanding the use of MWCNT for LSPR sensing of chemical and bio species. The developed Localized Surface Plasmon Resonant MWCNT bundles through *in-situ* synthesis of Au nanoparticles on MWCNT, is demonstrated by using LSPR technique as illustrated schematically in Fig. 1 for the detection of the bovine growth hormone . Herein, the CNTs are decorated with gold nanoparticles and the properties of the nanocomposites were explored to adsorb and detect the interaction of antibody-antigen through Localised Surface Plasmon Resonance.

Multicomponent assemblies such as nanotube/nanoparticle hybrids are a central part of next generation optical platforms for enhanced chemical and biological sensing. The nanostructures that integrate one-dimensional carbon nanotubes (CNTs) with zero-dimensional gold nanoparticles have numerous application potentials including the fabrication of nanodevices for chemical and biological sensors, cell imaging and drug delivery tools, supports for catalysts, inkjet printing, etc. The combination of their properties and dimensions leads to cooperative effects making the gold nanocomposites an ideal building blocks for molecular electronics as well^12^,^16^,^17^,^18^,^19^,^20^. The Au-CNTs nanocomposite films became an exciting field of research due to their inherent nature, influencing simultaneously the physical, chemical, electrical and optical properties due to the controlled integration of gold nanoparticles on CNTs.

Au-MWCNT nanocomposites are generally classified, according to the way gold is attached: without linker molecules and linked nanocomposites, where gold is linked, either covalently (for example through Au-S bonds), or non-covalently, by π-π stacking, hydrophobic forces or electrostatic interactions^21^,^22^. Pyrene derivatives are used as interlinkers because the chromophore of the molecules can be attached by π-π stacking interactions to the sidewalls of MWCNTs, while their alkylamine groups bind covalently to gold nanoparticles^23^ Gold nanoparticles can be attached onto the surface of sidewalls and ends of thiol-terminated multi-wall carbon nanotubes (MWCNTs) by using an aromatic linker^24^ or dithiol- and amino interlinkers^25^.

Various approaches have been reported to synthesize Au-CNT hybrid materials by using both the single wall carbon nanotubes (SWCNTs) and MWCNTs. They include solid state reactions at high temperatures in an inert atmosphere^26^, physical evaporation (thermal and e-beam)^27^, electrodeless deposition from the corresponding salt solutions, microwave assisted deposition, electrochemical reduction, galvanic displacement reaction, use of reducing agents (sodium borohydride, sodium citrate, ethylene glycol) or/and of different surface coupling chemistries^28^,^29^,^30^,^31^, anchoring functional groups and polymer surfactants as both template and dispersant, electrochemical reduction, etc.^16^,^32^. Gold nanoparticle filled CNTs were obtained by heat treatment with ammonia as well^21^.

The low redox potential of carbon nanotubes allows the spontaneous formation of gold and platinum nanoparticles on their surface, without the need of using a reducing agent^33^. The spontaneous formation of gold nanoparticles on the surface of CNTs was generally carried out by depositing the precursor solution directly on the surface of CNTs grown on a silica substrate^34^. Qu *et al.* used a metal (Cu) substrate to hold the CNTs and provide the electrons^26^,^35^,^36^ and negatively charged carbon nanotubes were also used to facilitate the reduction^37^. Recently, a hydrothermal method was reported, where the reduction is considered to take place spontaneously but actually, it had to be activated by heating under pressure in an autoclave. In addition, the time of the reaction was long and the gold particles formed on the tubes, were found to be very large (500 nm)^36^. *In situ* reduction of Au^3+^ on functionalized SWCNTs has been reported as well and the process was called “self-reduction”^38^.

Gold-MWCNTs have been synthesized until now by debundling the tubes, mostly through prolonged sonication. In the present work, we report on the fabrication of gold-decorated MWCNTs by the reduction of Au^3+^ in hydrogen chloroauric acid (AuHCl_4_) aqueous solution, on the surface of non-dispersed (or partially dispersed) MWCNT bundles. Bundles are complex entities where the MWCNTs’ collective properties manifest themselves in a new way. They are networks of self-assembled MWCNTs, with different sizes and shapes, where the tubes are held together by strong Van der Waals forces.

The aim of this work is to study the formation of the nanocomposite through the reduction of gold ions at the contact areas between the bundle of MWCNTs and the gold precursor during the evaporative migration of a casted drop on a glass substrate. After the drying of the drop, a so-called “coffee ring” pattern is formed on the glass, with the gold nanoparticles accumulated along the perimeter of the dried drop. The size distribution of Au nanoparticles and aggregates on the tubes are studied as a function of the mixing time and the concentration of the precursor solution. The reduction reaction is investigated at low temperature, in the ‘frozen’ state of the system as well, in the vacuum chamber of the Scanning Electron Microscope. The MWCNTs used in this work are pristine, i.e., not pre-treated with concentrated acids for the activation of their wall surfaces. The low temperature behavior of the MWCNTs – hydrogen chloroauric system is investigated in this work for the first time. The morphology and the optical properties of Au-MWCNT hybrids prepared through the ‘coffee ring’ method are studied by SEM and EDS, UV-Visible spectroscopy, Raman spectroscopy and imaging.

## Materials and Methods

**Figure 2:**
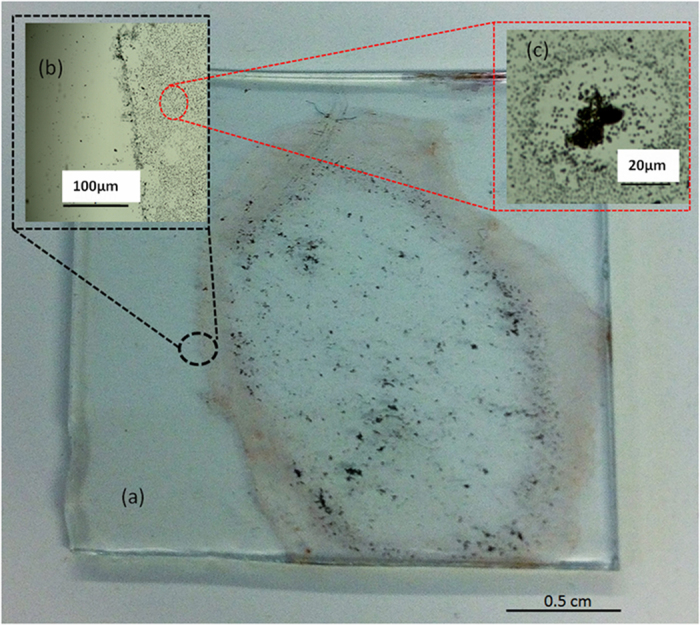
“Coffee ring” pattern of the dried drop on a glass substrate. Photograph of the dried drop (**a**), Optical micrograph image of the perimeter of the ring (**b**), and the enlarged image of a micro ring in the inner area of the ring (**c**).

For the room temperature experiments, a solution of HAuCl_4_ (~10^−4^ mole %) was mixed with a MWCNT (15–25 mg in 10 mL DI water) and, after mixing manually for around 10 min, it was kept for 30 min in an ultrasound bath (Branson 5510, 40 Khz, Danbury, CT, USA). After letting the mixture to stay for 10 min, one or more drops of the mixture were deposited on a glass substrate. The “coffee ring” pattern formed on the substrate after drying is shown in Fig. 2.

For the low-temperature experiments, aqueous solutions of gold chloride having concentrations in the range of 1.0 × 10^−4^–4.0 × 10^−4^ mol %, were prepared in DI water. Separately, MWCNTs (10 mg) were suspended in 10 mL DI water, without using any surfactant and without sonication. The process is explained in the following steps; (i) A weighing plastic dish (Part#: SKU GS3620, Fisher scientific) was cooled slowly by adding liquid nitrogen to it. This precooling is important to freeze the gold chloride solution effectively. (ii) 5 ml of gold chloride solution was added to precooled dish. (iii) Add slowly liquid nitrogen to the dish so that the gold chloride solution freezes instantaneously and a layer on the dish. (iv) 5 ml of MWCNT solution in DI water was added on top of gold precursor frozen layer. (v) Add slowly the liquid nitrogen to the dish so that the solution freezes and form another layer on the top. (vi) The weighing dish with two frozen layers of gold chloride solution and MWCNT solution was introduced into SEM chamber instantaneously for the measurements. The freezing of layers ensures that no reduction occurs before starting the measurements.

Images as well as EDS measurements for the quantification of gold were taken sequentially during more than two hours.

UV-Visible spectra of the composites were recorded by using the Perkin-Elmer spectrometer (Model 650) and the Raman spectra were measured with a Renishaw system by using an Ar ion (514.5 nm) laser.

### Experimental protocol for biosensing experiments

**Figure 3:**
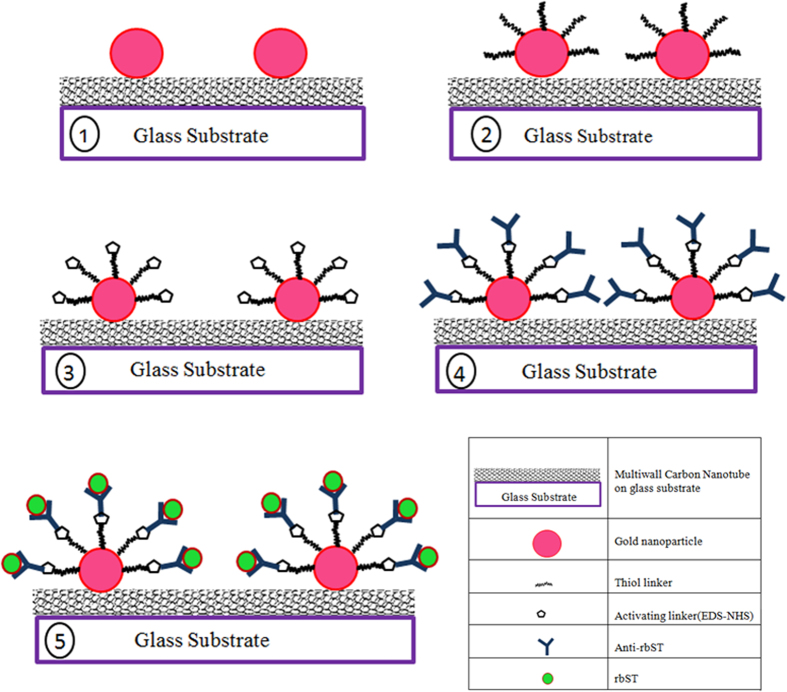
Biosensing protocol using Au-MWCNT nanocomposite as a sensing platform (Step 1: Au-MWCNT deposited on the glass substrate, Step 2: Au nanoparticles with a thiol linker, Step 3: Activating linker (EDS-NHS), Step 4: Anti-rbST is attached to gold through the linker, and Step 5: RbST (antigen) is attached to the antibody.

A schematic, explaining the various steps in the biosensing protocol is shown in Fig. 3. The sensor chip with the gold-MWCNT nanocomposite is shown in the first step. Then, few drops (0.5 ml) of a linker solution (HS (CH_2_) _n_-COOH) are placed on the sample, left for 2 hours (Step2 in Fig. 3) and then washed with a PBS solution. Then, the linker was activated by adding a solution containing a mixture (1:1) of EDC and NHS on the whole surface of the sample and keeping for an hour (Step 3 in Fig. 3). In the step 4, anti-rbST was adsorbed on to the sample and kept for one hour. The UV-Visible absorbance spectrum was recorded at this stage and, then the rbST was added to the sample (Step 5 in Fig. 3) and let to be absorbed on the sample for one hour. The UV Visible spectrum was measured after Step 5 again and the shift of the peak from Step 4 to Step 5 is measured as a measure of antibody-antigen interaction. The sensing experiments conducted are fully controlled with known concentration of pure rbST and anti-rbST.

## Results and Discussion

### Formation of the Au-MWCNT nanocomposite at room temperature

**Figure 4:**
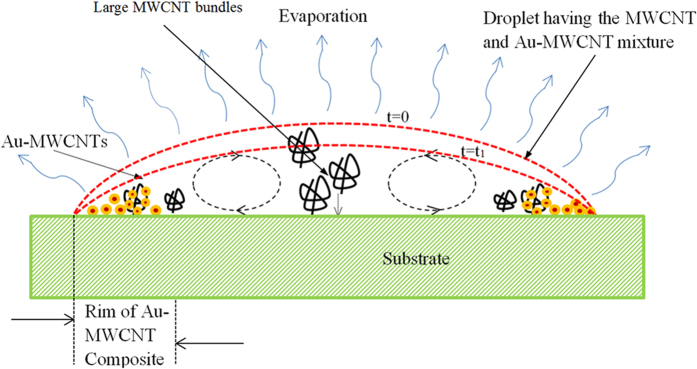
Cross section of a drop during evaporation. Formation of coffee-ring pattern of gold, MWCNT bundles and Au-MWCNTs composite on glass substrate.

The “coffee ring” pattern formed through the slow drying of the aqueous mixture casted on the substrate is shown in Fig. 4. It can be seen that at the edge, there are two adjacent zones: the outer region shows an accumulation of gold nanoparticles, while in the inner one, the MWCNTs appear to predominate. Larger bundles are visible in the central area as well and they are surrounded by gold nanoparticles as shown in Fig. 2(c). The whole pattern indicates multiple regions where gold nanoparticles are located in the proximity of the bundles. When several small drops are casted on the substrate, multiple intersecting coffee rings are formed, each of them showing the same pattern. It can be inferred that their nucleation and growth takes place in areas where the gold precursor molecules are carried by the evaporative flux of water in the vicinity of the bundles and/or over them. The general mechanism of the formation of coffee rings was described by Deegan^39^ and, recently, the phenomenon was successfully used for the fabrication of transparent conductive coatings, using colloidal solutions of gold or silver nanoparticles^40^,^41^.

MWCNT bundles are aggregates of tubes held together through Van der Waals forces. When exposed to the gold precursor solution, the reduction of gold ions results in the instantaneous formation of gold nanoparticles on the outer surface of the bundle.

As shown in Fig. 4, there are two concentric rings at the periphery gold the outer and MWCNTs in the inner one. It is thought that the nanocomposite is formed by their interaction at the interface as well in other areas inside the ring, where they come in contact during the evaporative migration. Figure 4 shows schematically the evaporation of a drop at two subsequent times (t = 0 and t = t_1_), together with the migration of the two entities towards the periphery of the drop. During the migration process, there will be a separation of the entities based on their different mass, accompanied by their deposition. The reaction for the formation of gold proceeds all along the migration and deposition. It is found that smaller MWCNT bundles, close to the edge of the drop, are more reactive towards the precursor molecules to form the gold. At the same time, large bundles will stay in the middle of the ring without forming the composite.

**Figure 5:**
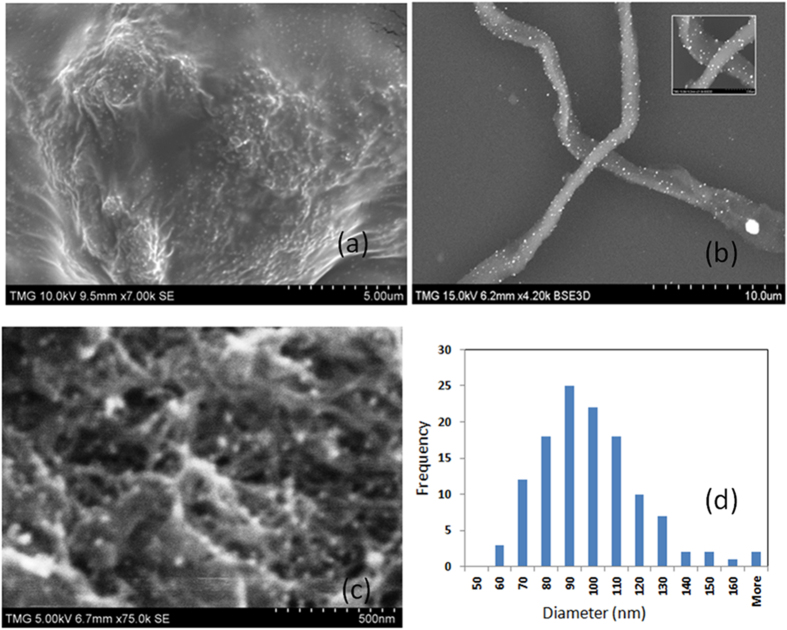
SEM image of a large bundle with gold nanoparticles attached to the branches (**a**), two intersected bundles with well-separated gold particles adsorbed on them (**b**), image, showing at high magnification, the network of tubes inside of the bundle (**c**), Size distributions of Au NPs (**d**). The concentration of the gold precursor in this case is low (~10^−4^ mole %) and the mixing time is short (1 min). The solution is kept for 30 min in the ultrasound bath.

The SEM image of bundles, loaded with gold nanoparticles, formed through the reduction of Au^3+^ during the drying of the drop is shown in Fig. 5(a–c). Figure 5a shows that bundles are quite large (here the diameter is around 20 μm) and they may enclose a huge amount of gold covered MWCNTs. The bundle in Fig. 5(a) appears to be “alighted” by the gold nanoparticles attached to the branches. The image shows clearly the closeness of the tubes and the Au nanoparticles on the short and long branches that are parts of the bundle. The two intersected bundles shown in Fig. 5(b) (1–2 μm diameter) confirm their distinct entities and show gold nanoparticles scattered on their outer surfaces. Fig. 5(c) shows the complex network of MWCNTs with the individual tubes crossing each other and the gold nanoparticles as white dots. The interaction of gold chloride solution with the ultrasound activated surface of MWCNT results in reduction and formation of gold nanoparticles on the surface of MWCNT. The image shows the closeness of the Au nanoparticles and the MWCNTs confirming that their nucleation and growth is taking place mostly on the surface due to the interaction with the MWCNT. The size of nanoparticles is found in the range of 80–100 nm as shown in the size distribution in Fig. 5(d).

**Figure 6:**
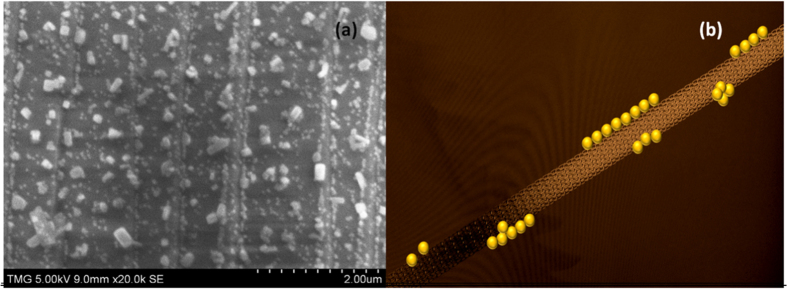
Linear aggregates of gold nanoparticles (**a**) Schematic of gold linear aggregates on the outer surface of the bundle (**b**).

When the contact time between the precursor solution and MWCNTs was increased to 5 min (*prior* the sonication) the images show the formation of different patterns of gold nanoparticles on the substrate (Fig. 6). There is a high coverage of discrete gold nanoparticles and linear aggregates in parallel rows, while larger non-spherical particles are distributed between the rows. The backbones of the aggregates are formed from several rows of spherical particles to which branched structures are attached.

It is known that gold nanoparticles, because of the strong affinity between them, often assemble in linear aggregates^42^. It is not clear, however, why the gold particles form parallel and approximately equidistant rows that seem to be in a higher plan compared to the plane of the substrate. The rows are framed alongside by larger non-spherical gold crystals. The formation of linear aggregates could be useful for the fabrication of nanowire structures and other nanoscale building blocks^24^. A simplified schematic of formation of gold linear aggregates is shown in Fig. 6(b).

### Raman spectroscopy and imaging of gold – MWCNT hybrids

**Figure 7:**
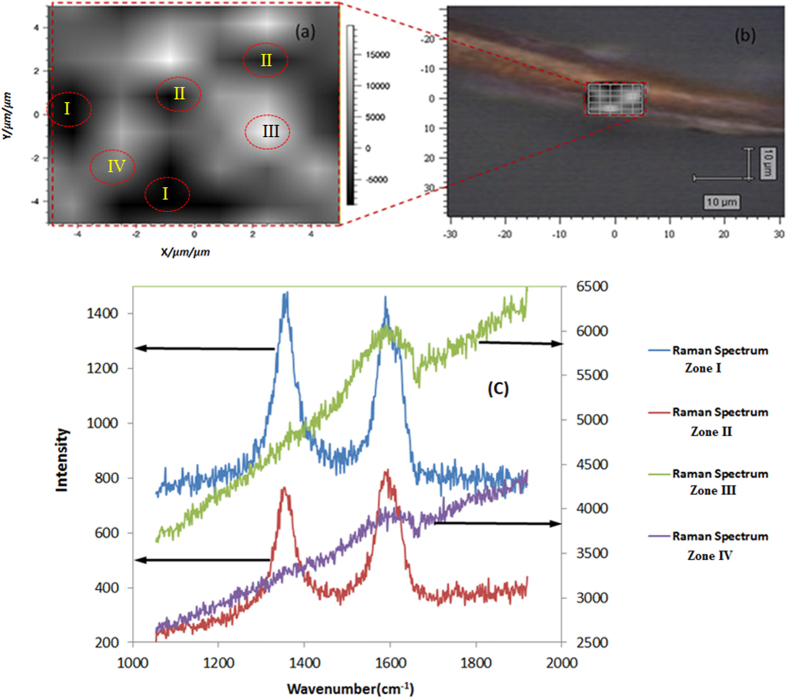
Combined video and Raman image of a MWCNT covered by aggregates of gold nanoparticles (**a**,**b**), and Raman spectra corresponding to measurements at different Zones on the sample (Zone I to Zone IV) of the area defined in (**a**) according to the mapping software (**c**). The Raman image (**a**) originates in the mapping of the ratio of the intensities of G (1590 cm^−1^) and D (1343 cm^−1^) lines. The bright spots in the Fig. 7(a) correspond to the high values of the intensity ratio, in the areas where the density of gold is the highest.

One could note from Fig. 7 a high areal density of gold particles and clusters on the walls of CNTs. The particles are not isolated, they form clusters and large aggregates, due to the small diffusion barriers and high interfacial energies^43^. Figure 7(c) shows the two main typical bands present in the Raman spectrum of Au-MWCNTs bundles: the band at 1580 cm^−1^ (G band) assigned to the in-plane vibration of the C–C bond, with a shoulder around 1604 cm^−1^, typical of defective graphite-like materials and the band at 1342 cm^−1^ (D band) activated by the presence of disorder in carbon systems. Compared to the bands of MWCNTs, the bands showed little change, indicating a weak interaction between the Au nanoparticles and carbon nanotubes. Figure 7(c) shows that the relative intensity of the two bands (I_1580_/I_1342_) depends on the area where the measurement is taken. While in some of the images there are two well-defined bands of almost the same intensity, in others, the 1342 cm^−1^ band cannot be seen. The apparent suppression of D band is related to a poor signal to noise ratio, probably due to the high concentration of gold at these locations (Zones 3 and 4) that reflect a large amount of excitation photons and acts as screening mirror preventing Raman scattering generation. This would support the fact that the spectra that originate from a high Au aggregation result in suppression of D-band and broadening of G-band due to reflecting effect of gold. The slope line could be due to fluorescence or scattering or luminescence of the gold clusters. No SERS effect has been observed in the case of the Au-MWCNT hybrids prepared in this work.

### Mechanism of the reaction at room temperature

**Figure 8:**
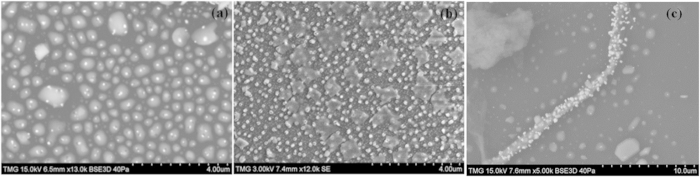
Nucleation of gold inside the dried drop of the precursor solution (**a**) and spreading over and growing of the gold crystals (**b**) and reduction of gold on CNT bundles.

The images of the dried gold precursor on the substrate where gold nanoparticles can be seen in their nascent form in Fig. 8. In image 8a, particles can be seen inside the precursor area, while in 8b, most of the particles are larger and they are outside the circular shapes. The two images are at the same scale and correspond to two different moments on the time scale of the reaction. It can be inferred from this succession of images that nuclei of gold are first formed at the contact area of the substrate and the drop of the precursor solution, during the evaporative migration of the mixture. The image (8a) shows that the majority of the circular shapes have only one nuclei of gold. Later on, the gold crystals are growing and spreading out over the whole surface. The EDS analysis indicated a concentration of gold of 6 weight % in the coffee ring pattern of the dried composite. We assume that either, on or under the surface, there is a bundle of tubes that will host the newly formed gold nanoparticles. In order to gain additional information on the kinetics of this reaction, low-temperature experiments have been performed and the evolution of the system -was followed *in-situ,* in the vacuum chamber of the SEM microscope.

### Low-temperature reaction between the gold precursor and MWCNTs

**Figure 9:**
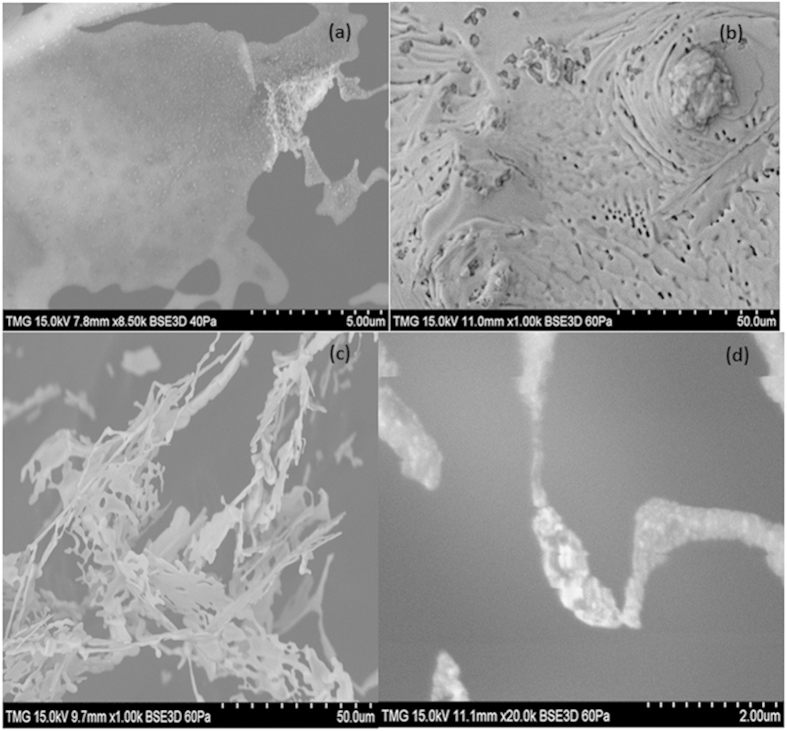
Frozen bundles spread over a large surface. Images were taken approximately at 30 min intervals, from the beginning of the experiment until the end (2 hours). Figure 9(a) corresponds to 30 min, (**b**) to 60 min, (**c**) to 90 min and (**d**) to 120 min from the beginning of the experiment. The temperature of the sample during the whole experiment was around 4–6 °C.

The temperature in the vacuum chamber of the instrument during the whole experiment was well below of the melting point of the HAuCl_4_ solution. The bundles of MWCNTs were embedded in the frozen solution of the precursor as shown in Fig. 9. Under these conditions, the rate of the reaction was considerably reduced. Gold nanoparticles can be seen in Fig. 9(a,b) and the quantification by EDS indicated the presence of quite a high concentration of gold as shown below. The concentration of gold in different areas varies between 20 and 28%. Au NPs can first be seen in images taken after 30 min from the beginning of the experiment. Figure 9 corresponds to the images of the sample taken at different time intervals. The Fig. 9(a–d) show the images of the sample after 30 min, 60 min, and 90 min respectively. The local melting of the frozen sample and gradual formation of gold nanoparticle can be seen in Fig. 9. The Fig. 9(d) shows the gold nanoparticle decorated MWCNT bundle. It means that, probably, there are some points of contact where the solution of the precursor starts to melt and the reaction can take place locally, on the surface of the bundle.

In spite of the low temperature, there is a high density of gold particles covering the surface of the bundles. It can be seen that the bundles, for example, in the right upper part of the image, are completely covered by the still frozen solution. This image confirms that, even at the end of the SEM experiment, that is, after more than two hours, the system is almost totally frozen. However, the small dark areas seen everywhere in the image show the beginning of the local melting processes that will accelerate the reaction.

The SEM images recorded chronologically during the low-temperature experiment prove, without any doubt, that gold ion can be reduced on the surface of bundles at temperatures as low as 4–6 °C to make the MWCNT locally sensitive to LSPR properties.

### Label-free biosensing of bovine growth hormone (bovine somatotropin (bST), based on the LSPR of gold nanoparticles

**Figure 10:**
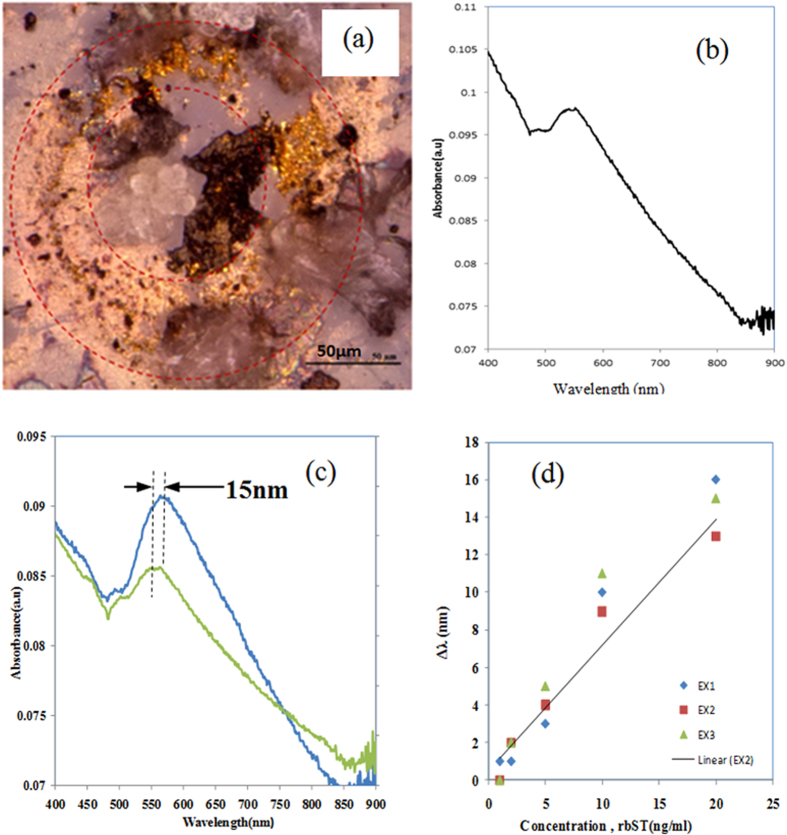
(**a**) optical microscope image of the Au-MWCNT sample with the immobilized antigen and antibody, The image shows a micro ring of nanocomposite surrounding a large bundle of MWCNTs. (**b**) Au LSPR corresponding to the Au-MWCNT composite, and (**c**) the change of the position of LSPR in the presence antibody (green) and, after the interaction with a solution of 20 ng/mL of antigen (blue). (**d**) Calibration curve of sensor with 3 experiments done for each concentration of 20 ng/ml, 10 ng/ml and 5 ng/ml.

The proposed Au-MWCNTs nanocomposite is an attractive candidate for the label-free sensing of proteins. In order to demonstrate the sensing potential of the nanocomposite, recombinant bovine somatotropin (rbST) is used for the experiments. The localized surface plasmon resonance (LSPR) property of the gold was measured as shown in Fig. 10(a). The absorbance peak of the nanocomposite is measured at 548 nm. In order to adsorb anti-rbST, a thiol group-ended surfactant was used as shown in detail in the experimental part.

An optimized protocol for immobilizing anti-rbST and rbST on gold nanostructures in our previous works^2^,^3^,^4^,^44^ was used in the present work (see Fig. 3). In the sensing experiments, the optical absorbance spectrum was measured during each step of the sensing protocol. First, the anti-rbST was immobilized on the gold in the nanocomposite. The LSPR spectrum corresponding to the sample having the adsorbed anti-rbST is shown in Fig. 10(c). The peak in the spectrum is shifted to 553 nm that is by 4 nm, upon adsorption of anti-rbST (20 ng/mL). Subsequently, rbST (20 ng/mL) was added to the sample. Upon the adsorption of rbST, the spectrum was further shifted to 569 nm, which corresponds to a LSPR shift of 16 nm, upon antigen-antibody interaction. In addition to this significant shift, indicating a strong antibody-antigen interaction, Fig. 10(c) shows an increase of the intensity as well. However, the plasmonic shift of Au LSPR shows a better linear dependency on the concentration. Figure 10(b) shows the microscope image of the sample with anti-rbST and rbST adsorbed in a micro-coffee ring structure. The experiments were repeated with concentrations of rbST between 0.5 ng/ml and 20 ng/ml. The results show that the sensor is giving repeatable results and the sensitivity of the platform is found to be very high compared with that of the gold nanoparticles alone. The LSPR sensing with only Au nano particles yielded a sensitivity of only 25 ng/ml so far^3^. But, the lowest concentration that could be detected with this Au-MWCNT platform is found to be 1 ng/ml of rbST, confirming that the Au-MWCNT provides an ultrasensitive platform for the plasmonic detection of polypeptides and proteins.

## Conclusion

The formation of the Au – MWCNT nanocomposite through *in situ* reduction of gold ions on the surface of bundles of MWCNTs is investigated under various conditions. It is demonstrated that the reaction takes place during the evaporative migration of the two entities on a glass substrate, after the mixture was activated by ultrasonic agitation. The nanocomposite is accumulated mostly at the contact area (interface) of the two rings (gold and MWCNTs), but also in the micro rings formed inside the coffee ring migration pattern. The nanocomposites are investigated by SEM and EDS, Raman and UV-Visible spectroscopy at room temperature as well as at low temperatures under vacuum, when the MWCNTs are embedded in the frozen solution of the gold precursor solution. The size of gold nanoparticles formed under these conditions is around 90–100 nm.

The growth of gold nanoparticles will result in gold crystals with various sizes and shapes and, in some areas, linear aggregates in parallel rows are formed. The sensing capability of the Au – MWCNT nanocomposite is demonstrated by using the gold plasmon band. The results showed a high sensitivity of the nanocomposite toward the optical detection of the growth hormone, bovine somatotropine.

## Additional Information

**How to cite this article**: Ozhikandathil, J. *et al.* Plasmonic Gold Decorated MWCNT Nanocomposite for Localized Plasmon Resonance Sensing. *Sci. Rep.* **5**, 13181; doi: 10.1038/srep13181 (2015).

## Figures and Tables

**Figure 1:**
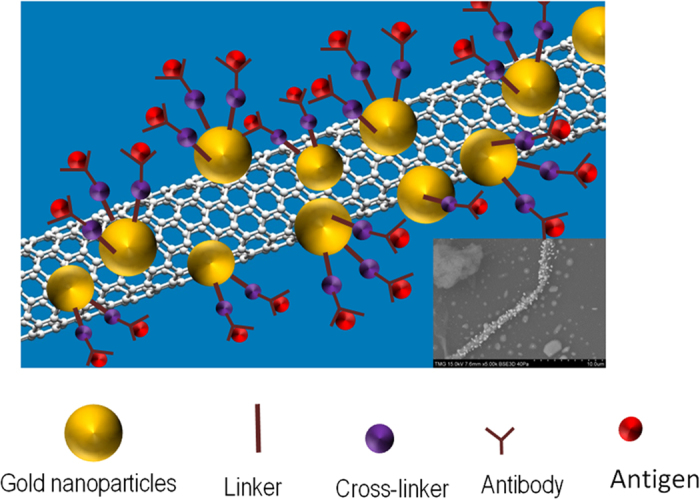
Carbon nanotubes locally decorated with gold nanoparticle for plasmonic detection. The SEM image of the Au-CNT is shown in inset.
